# Upconverting nanoparticle reporter–based highly sensitive rapid lateral flow immunoassay for hepatitis B virus surface antigen

**DOI:** 10.1007/s00216-020-03055-z

**Published:** 2020-11-23

**Authors:** Iida Martiskainen, Sheikh M. Talha, Karoliina Vuorenpää, Teppo Salminen, Etvi Juntunen, Souvick Chattopadhyay, Dinesh Kumar, Tytti Vuorinen, Kim Pettersson, Navin Khanna, Gaurav Batra

**Affiliations:** 1 Department of Biotechnology, University of Turku, 20014 Turku, Finland; 2 Translational Health Science and Technology Institute, NCR Biotech Science Cluster, Faridabad, 121001 India; 3 Arrow weighing systems Pvt Ltd (unit Designinnova), New Delhi, 110028 India; 4 Department of Virology and Clinical Microbiology, University of Turku, 20520 Turku, Finland; 5 International Centre for Genetic Engineering & Biotechnology, New Delhi, 110067 India

**Keywords:** HBsAg, HBV diagnostics, Upconverting nanophosphors, Lateral flow, Point-of-care test

## Abstract

Detection of hepatitis B Virus surface antigen (HBsAg) is an established method for diagnosing both acute and chronic hepatitis B virus (HBV) infection. In addition to enzyme immunoassays (EIAs), rapid diagnostic tests (RDTs) are available for the detection of HBsAg in resource-poor settings. However, the available RDTs have inadequate sensitivity and therefore are not suitable for diagnosis of patients with low levels of HBsAg and for blood screening. To provide a high-sensitivity RDT, we developed a lateral flow immunoassay (LFIA) for HBsAg utilizing upconverting nanoparticle (UCNP) reporter. The UCNP-LFIA can use whole blood, serum, or plasma and the results can be read in 30 min using a reader device. When compared with a commercial conventional visually read LFIA, the developed UCNP-LFIA had a Limit of Detection (LoD) of 0.1 IU HBsAg/ml in spiked serum, whereas the LoD of the conventional LFIA was 3.2 IU HBsAg/ml. The developed UCNP-LFIA fulfills the WHO criterion for blood screening (LoD ≤ 0.13 IU HBsAg/ml) in terms of LoD. The UCNP-LFIA and conventional LFIA were evaluated with well-characterized sample panels. The UCNP-LFIA detected 20/24 HBsAg-positive samples within the HBsAg Performance Panel and 8/10 samples within the Mixed Titer Performance Panel, whereas the conventional LFIA detected 8/24 and 4/10 samples in these panels, respectively. The performance of the assays was further evaluated with HBsAg-positive (*n* = 108) and HBsAg-negative (*n* = 315) patient samples. In comparison with a central laboratory test, UCNP-LFIA showed 95.4% (95% CI: 89.5–98.5%) sensitivity whereas sensitivity of the conventional LFIA was 87.7% (95%CI: 79.9–93.3%).

**Supplementary Information:**

The online version contains supplementary material available at 10.1007/s00216-020-03055-z.

## Introduction

Hepatitis B virus (HBV) can be transmitted through contact with infected blood or other body fluids as well as perinatally from infected mothers to neonates. There are around 257 million persons worldwide living with HBV, and as per 2015 data, HBV infection resulted in around 887,000 deaths [1, 2].

HBV is a major health problem in low- and middle-income countries (LMIC) [3]. Worldwide, over 67% of chronically infected carriers of HBV are living in Asia and Africa. Prevalence is highest in the African (6.1%) and Western Pacific regions (6.2%) [1]. Many countries in these regions have HBV prevalence higher than 10%.

Most of the infected individuals are asymptomatic at the early stage of infection. However, a small proportion of acute infections are symptomatic, and a very small proportion can develop acute liver failure. In some individuals, HBV can cause chronic liver infection which may result in cirrhosis and hepatocellular carcinoma leading to death [4].

Identification and treatment of asymptomatic people with chronic HBV infection should reduce the disease burden. However, HBV screening is non-existent in several LMIC and even the blood donor screening is performed using tests with inadequate sensitivity [5]. Only 10.5% of all chronically infected individuals are aware of their infection [2].

The recommended and most used method for the screening of HBV is serological testing of HBsAg [6]. Highly sensitive enzyme immunoassays (EIAs) can be used for reliable detection of HBsAg. However, EIAs require sophisticated laboratory infrastructure with trained personnel and have turn-around-times ranging from 3 to 5 h. Moreover, because of the time gap between sampling and results, follow-up with the patient is often at risk. Rather than EIAs, rapid diagnostic tests (RDTs) like lateral flow immunoassays (LFIAs) provide an alternative for the detection of HBsAg as the RDTs can be used without laboratory infrastructure with minimal training. The greatest advantage of RDTs is their cost-efficiency and the turn-around-times of 15 to 30 min.

RDTs are used for diagnostic purposes and even for the screening of blood donors in many resource-limited settings when EIAs are not feasible [7]. However, the performance of the RDTs used for the detection of HBsAg is often inferior compared with central laboratory tests. In Ghana, blood donor screening for HBsAg using RDT with a sensitivity of 7.5 IU/ml revealed 8% prevalence. In the same population, a sensitive EIA with analytical sensitivity of 0.05 IU/ml HBsAg revealed a prevalence of 14–15% [3]. The available data shows that the analytical sensitivity of most RDTs is in the range of 2–10 IU/mL which is approximately 50- to 100-fold less sensitive compared with widely used CE-marked EIAs [8, 9].

Screening of blood products for transfusion requires high-sensitivity assays to detect low HBsAg levels for, e.g., those found in early seroconversion to minimize the risk of transfusion transmitted infection [9]. As per the World Health Organization (WHO) performance evaluation acceptance criteria for HBsAg test in the context of WHO Prequalification, an assay should have analytical sensitivity of ≤ 4 IU/mL to be used for routine diagnostic purposes and ≤ 0.13 IU/ml for the screening of blood donations [9].

The inadequate sensitivity of the typical lateral flow tests is affected by the relatively short incubation time for antigen and antibody interaction [10] and the inadequate signal generation strength of traditionally used visual labels such as colloidal gold and carbon nanoparticles [11]. Moreover, visual readout is associated with a risk of misinterpretation of the results particularly with equivocal samples. The subjectivity involved with interpretation of visual results can be removed by using a suitable reader device. However, use of a reader device alone does not improve the sensitivity of the assay. It has been reported earlier that the sensitivity of lateral flow assays can be improved by replacing the visual labels with fluorescent nanoparticle reporters [12, 13].

Upconverting nanoparticles (UCNP) have successfully been used as reporters in the development of highly sensitive lateral flow assays for the detection of wide range of target analytes including *Schistosoma* circulating anodic antigen [14], hCG [15], IL-10 [16], cTnI [17], prostate-specific antigen [18], ephrin type-A receptor 2 [18], HBV nucleic acids, small molecules such as ochratoxin A, heavy metal ions (Hg2+), and bacteria such as *Salmonella* [19]. Also, UCNP-LFIAs have been used for quantifying myoglobin [20] and for the measurement of anti-HBV surface antigen antibodies [21] among others.

UCNPs have a unique feature of converting lower energy excitation wavelength into higher energy emission at visible wavelengths [22]. Use of UCNP reporters can further enhance the assay sensitivity compared with traditional fluorescent labels since it eliminates the measurement background autofluorescence originating from the sample matrix or the components of the test device. This background elimination is due to the spectral separation of UCNP excitation, emission, and background autofluorescence wavelengths [23].

Here, we designed and produced a UCNP reporter–based LFIA (UCNP-LFIA) for high-sensitivity detection of HBsAg in serum, plasma, and whole blood. First, we generated monoclonal antibodies (mAbs) against HBsAg, selected the best pair of mAbs, and optimized the UCNP-LFIA components and assay conditions. The developed assay was evaluated using well-characterized panels of clinical samples, WHO International standard for HBsAg, genotype panels, and a seroconversion panel. In addition, the results were compared with a commercially available conventional HBsAg LFIA. UCNP-LFIA could achieve analytical sensitivity of 0.1 IU HBsAg/ml in serum compared with 3.2 IU HBsAg/ml of the conventional LFIA.

## Materials and methods

### Samples and reference assays

WHO Third International Standard for HBsAg (12/226) was purchased from the National Institute for Biological Standards and Controls (NIBSC, United Kingdom). The 1st WHO International Reference Panel for Hepatitis B Virus (HBV) Genotypes for Hepatitis B Surface Antigen (HBsAg) Assays (6100/09) was purchased from Paul-Ehrlich Institute (Germany).

The following commercial sample panels were purchased from SeraCare Life Sciences Inc. (MA, USA): AccuSet™ HBsAg Performance Panel (0805-0340), AccuSet™ HBsAg Mixed Titer Performance Panel PHA207(M) (0805-0217), AccuSet™ HBV Worldwide Performance Panel (0805-0313), HBV Seroconversion Panel PHM941 (0606-0060) and 24 HBsAg-positive disease-state samples. These panels consist of undiluted, naturally occurring plasma samples. Fifty HBsAg-positive serum samples were purchased from Biomex GmbH (Germany). Eighteen HBsAg-positive plasma samples were purchased from Labquality Oy (Finland). Whole blood and serum used for international standard’s dilutions, as well as a sample panel consisting of 100 presumed HBsAg-negative serum samples, were purchased from Turku University of Applied Sciences (Finland).

Apart from commercially available sample panels, 215 clinical serum and plasma samples that tested negative for HBsAg and 16 samples that tested positive for HBsAg were obtained from the Department of Virology, University of Turku (Finland). All the patient data, except for the status for the presence or absence of HBsAg, were anonymized and no clinical data of the patients were handled. Use of these samples in this study was approved by the Ethical Committee of the Hospital District of Southwest Finland (Decision T012/011/18). The details of the reference tests used for the characterization of these samples are summarized in Table S1 (see Electronic Supplementary Material, ESM). Assay principle for the central laboratory reference tests is described in the ESM.

Apart from using central laboratory tests as reference, a commercially available conventional rapid LFIA (Alere DetermineTM HBsAg (code # 7D2543), Abbott Laboratories, USA) based on visual detection of signals was used for comparison.

### Anti-HBsAg antibodies

Mouse monoclonal antibodies against HBsAg were generated using hybridoma technology [24].

### Bioconjugation of UCNP reporters

Carboxylated Upcon™ UCNP reporter particles of 68-nm diameter with a hydrophilic coating (Kaivogen Oy, Finland) were covalently coupled with an in-house mouse anti-HBsAg monoclonal antibody (mAb) 3D3. A solution containing 1 mg UCNPs was centrifuged, the supernatant was removed, and the particle surface was activated by suspending the pellet into particle activation buffer (20 mM MES pH 6.1, 2 mM KF, 20 mM EDC, and 30 mM sulfo-NHS). The incubation was performed with rotation for 15 min at room temperature. The UCNPs were washed by centrifugation, removing the supernatant and suspending the UCNPs to 20 mM MES, pH 6.1. The UCNPs were centrifuged as before and resuspended into 20 mM MES, pH 6.1 containing 84 μg of mAb 3D3. This conjugation reaction was incubated for 2.5 h at room temperature with rotation and the reaction was stopped by adding glycine, pH 11. To remove non-covalently associated mAb, the UCNP conjugates were washed twice by centrifugation and the pellet was suspended in 500 μl of storage buffer (5 mM Tris, 0.05% NaN3, 0.05% Tween-85, pH 8.5). This step was repeated twice, and finally, the pellet was suspended to 250 μl of storage buffer and BSA was added to a final concentration of 0.5%.

### Preparation of LFIA strips

A lateral flow card was assembled on a plastic backing by pasting nitrocellulose membrane 200CNPH-N-SS60 (Advanced Microdevices Pvt. Ltd., India), a cellulose absorbent pad (CFSP223000, Merck Millipore, MA, USA), a glass fiber conjugate pad (8951, Ahlstrom-Munksjö Oyj, Finland), and a red blood cell separator sample pad (FR1 0.6, Advanced Microdevices Pvt. Ltd., India). The sample pad was pre-treated with blocking buffer solution (10 mM Tris-HCl pH 8.5, 135 mM NaCl, 0.5% Tween-20, 0.1% Triton-X-100, 0.8 mg/ml mouse IgG, 0.2% denatured mouse IgG, 0.24% bovine IgG). The test line solution consisted of equal proportions of three anti-HBsAg mAbs. Two of these mAbs were in-house (mAb 3G8 and mAb 4G9) and one was procured from a commercial source (mAb 2508 SPTN-5, Medix Biochemica Oy Ab, Finland). The line dispensing was performed in 10 mM Tris-HCl, pH 8.0 buffer on the nitrocellulose membrane at a concentration of 300 ng/cm. The control line was printed 4 mm from the test line with 300 ng/cm of rabbit anti-mouse IgG (Dako Products, Agilent Technologies Inc., USA). After printing test and control lines, the cards were dried overnight at + 35 °C, other components of the test strips were assembled, and the cards were stored at room temperature protected from humidity. The cards were covered with transparent cover tape (KN-CPP1-Clear Kenosha cover plastic, Kenosha, Netherlands) starting from the conjugate pad to the end of the absorbent pad. Before the use, the cards were cut into 4.8-mm-wide strips. Twenty nanograms of UCNP reporters were dried onto the conjugate pad of each of the strip in 10 mM Tris-HCl buffer, pH 8.5 containing 135 mM NaCl, 0.5% Tween-20, 1% BSA, 5% sucrose. The strips were dried protected from humidity. The strips were placed in a suitable plastic housing with a common inlet for sample and buffer.

### UCNP-LFIA procedure

To start the procedure, 50 μl of the sample was added into the sample inlet, followed by applying 50 μl of chase buffer (10 mM Tris-HCl pH 8.5, 135 mM NaCl, 0.5% Tween-20, 1% BSA, and 0.1% Triton-X-100). After 30 min, the test and control line upconversion photoluminescence signals were measured with an Upcon reader device (Labrox Oy, Finland) with excitation at 976 nm and emission at 550 nm. Details of the benchtop UCNP reader and a battery-operated portable UCNP reader are provided in the ESM (Fig. S10).

The interpretation of the results was based on the maximum signal measured at the test line position. The overall baseline signal measured along the strip was subtracted from the test line maximum signal. Thus, only the test line peak signal was considered the outcome of the measurement. The control line signals were interpreted as qualitative control.

### Limit of detection

Two methods were used for determining the limit of detection (LoD), both in serum and in whole blood. In the first method, dilutions of the WHO Third International Standard for HBsAg between 0.01 and 12.8 IU/ml were used for plotting standard curves. For the standard curve, 20 replicates were used for each of the three low concentration dilutions (0.05, 0.1, and 0.2 IU/ml) near the pre-estimated detection limit. Four replicate strips were used for the other concentrations.

The cutoff level for the LoD was determined by using 60 replicates of the blank sample and selecting the highest measured signal value from these replicates as the cutoff value. The LoD (IU/ml) was calculated from the cutoff and the equation obtained with linear regression of the standard curve.

The LoD was further confirmed with a second method by performing an additional test using 80 replicates of the three concentrations below, above, and at the same level (0.05, 0.1, and 0.2 IU/ml) as the previously calculated LoD. The sample size of 80 was based on a statistical sampling plan (ISO 2859-1:1999) previously used by Das et al. [25]. Sample size code J was determined by using general inspection level II and was based on a lot size of 501–1200. A single sampling plan for normal inspection was used, which resulted in a sample size of 80.

The LoD of conventional HBsAg LFIA (Alere DetermineTM HBsAg) was determined similarly with the WHO third international standard for HBsAg. The standard was diluted in serum in concentrations of 0.8, 1.6, 3.2, 4.8, 6.4, and 12.8 IU/ml. Dilutions were run on the commercial RDT, in 4 replicates for concentrations 0.8–6.4 IU/ml, and two replicates for concentration 12.8 IU/ml, according to the manufacturer’s instructions. The visual results were interpreted by two individuals.

### Assay performance evaluation

All the clinical samples and sample panels were evaluated with the developed assay according to the UCNP-LFIA procedure. All the clinical serum and plasma samples from different individuals not belonging to a commercial fixed sample panel (e.g., performance panel, mixed titer panel) were used for determining the sensitivity and specificity of the assay. The total sample numbers used for the calculation of the assay performance were 108 HBsAg-positive and 315 HBsAg-negative samples.

The optimal clinical cutoff value was determined based on receiver operating characteristic (ROC) analysis executed by SAS JMP Pro 14 statistics software. To calculate the signal-to-cutoff (S/Co), photoluminescence signal obtained from each sample with peak detection was divided by the cutoff. Samples with S/Co values ≥ 1 were considered reactive.

In order to compare the performance of the developed assay with a conventional HBsAg rapid LFIA, all the clinical samples were tested with commercial RDT according to the manufacturer’s instructions. The visual results were interpreted by two individuals who were blinded for the reference assay results.

### UCNP-LFIA strip stability study

The strips were stored in sealed aluminum foil pouches with a desiccant at + 37 °C for 50 days. The strip stability was studied at 7 time points during this period by testing the strips with the UCNP-LFIA procedure. Native HBsAg protein (Yashraj Biotechnology Ltd., India) was diluted in HBsAg-negative human serum in concentrations of 0, 0.5, and 5 ng/ml and used as a sample.

### Photoluminescence measurement reproducibility

Dry strips assayed with human serum spiked with 0, 0.5, and 5 ng/ml HBsAg were measured 15 times. The measurement reproducibility was determined by calculating the coefficient of variation between repeat measurements of the same strip.

## Results and discussion

**Fig. 1:**
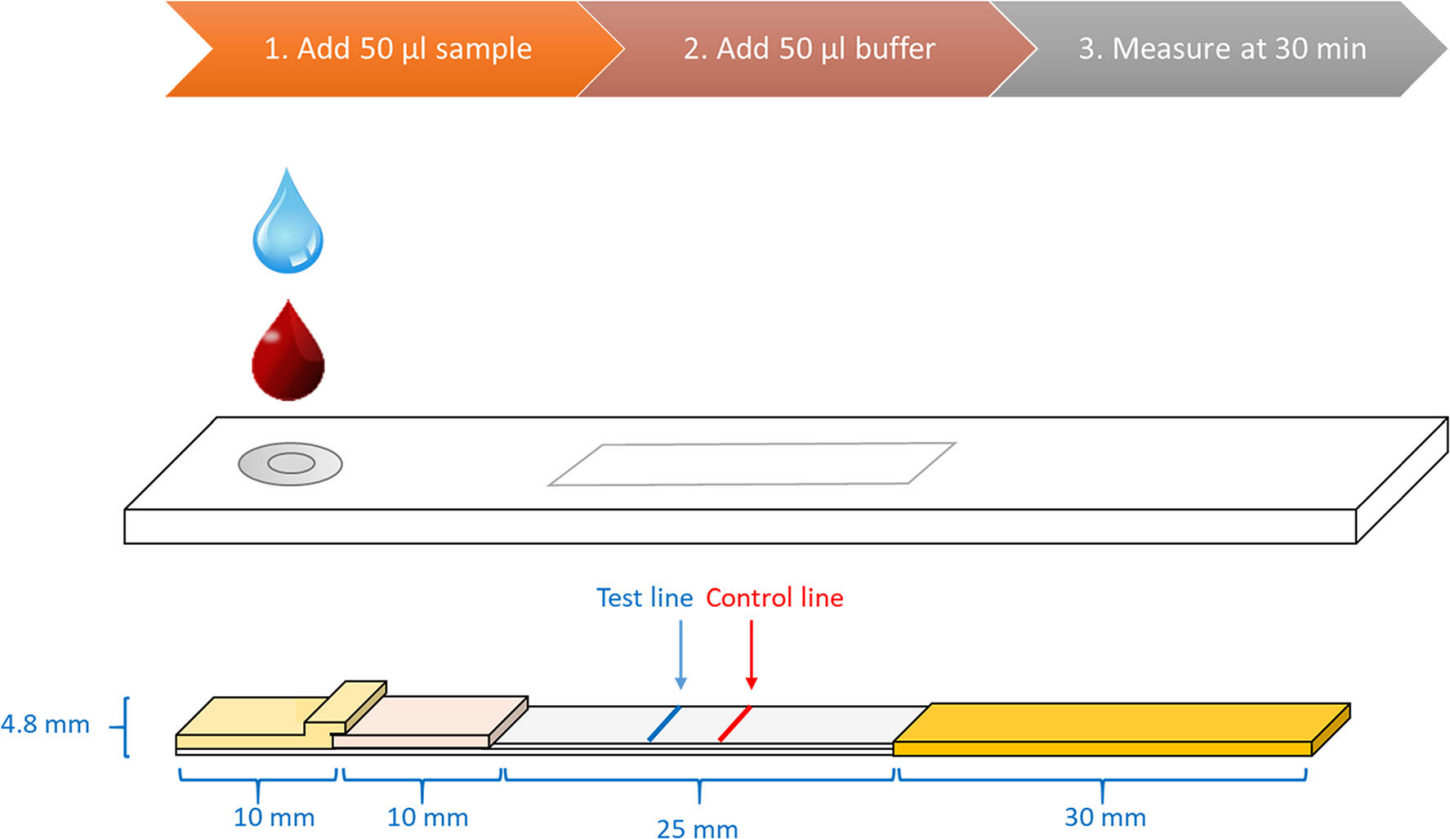
UCNP-LFIA strip design and assay procedure. The strip consisted, from left to right, of a sample pad, a conjugate release pad, a nitrocellulose analytical membrane, and an absorbent pad inside a plastic housing. The assay procedure was started by adding 50 μl of sample to the sample pad followed by 50 μl of chase buffer. The UCNP signal was measured after 30 min with a reader device. UCNP signal profile of strips is provided in ESM (Fig. S11)

The objective of the work was to develop an ultra-sensitive RDT that can bridge the sensitivity gap between conventional RDTs and the more complicated and resource-demanding EIAs for the detection of HBsAg. The developed RDT was a LFIA with a simple assay procedure (Fig. 1).

### Design and development of UCNP-LFIA for HBsAg

We developed the LFIA using UCNP reporter particles for the detection of HBsAg. The assay development and optimization included hybridoma antibody generation (ESM Fig. S1), identification of most optimal conjugate antibody (ESM Fig. S2), epitope binning (ESM Fig. S3, Table S2), and antibody screening based on the binding with different HBV genotypes (ESM Fig. S4), identification of suitable antibody pair for the UCNP-LFIA (ESM Figs. S2, S5, and Table S3), drying of the UCNP conjugates (ESM Fig. S6), identification of nitrocellulose membrane and optimal time of measurement (ESM Table S4, Fig. S7), and sample volume optimization (ESM Fig. S8).

The obtained data suggested that mAb 3D3 as tracer in combination with mAbs 3G8, 2508, and 4G9 as capture was the most optimal antibody combination for the UCNP-LFIA. The mAb 3D3 was chosen as tracer mAb based on signal and background levels obtained in the UCNP-LFIA, and also based on the conjugate colloidal properties. Based on epitope binning and evaluation of the genotype detection, we chose three non-competing capture antibodies. The antibodies were chosen based on their likelihood of binding to different epitopes on HBsAg (ESM Fig. S5) in order to reduce the risk of UCNP-LFIA missing HBsAg mutants. The MAb 3D3-UCNP conjugate was dried to the conjugate pad during the assay development phase. No difference between dry and liquid conjugates was observed (ESM Fig. S6). Optimal nitrocellulose membrane (CNPH-N SS60) and read time (30 min) were chosen based on the maximum signal difference between positive and negative spiked whole blood samples (ESM Fig. S7). An increase in sample volume to 50 μl resulted in higher specific signals (ESM Fig. S8) and was chosen for further work.

### Determination of the limit of detection

**Fig. 2:**
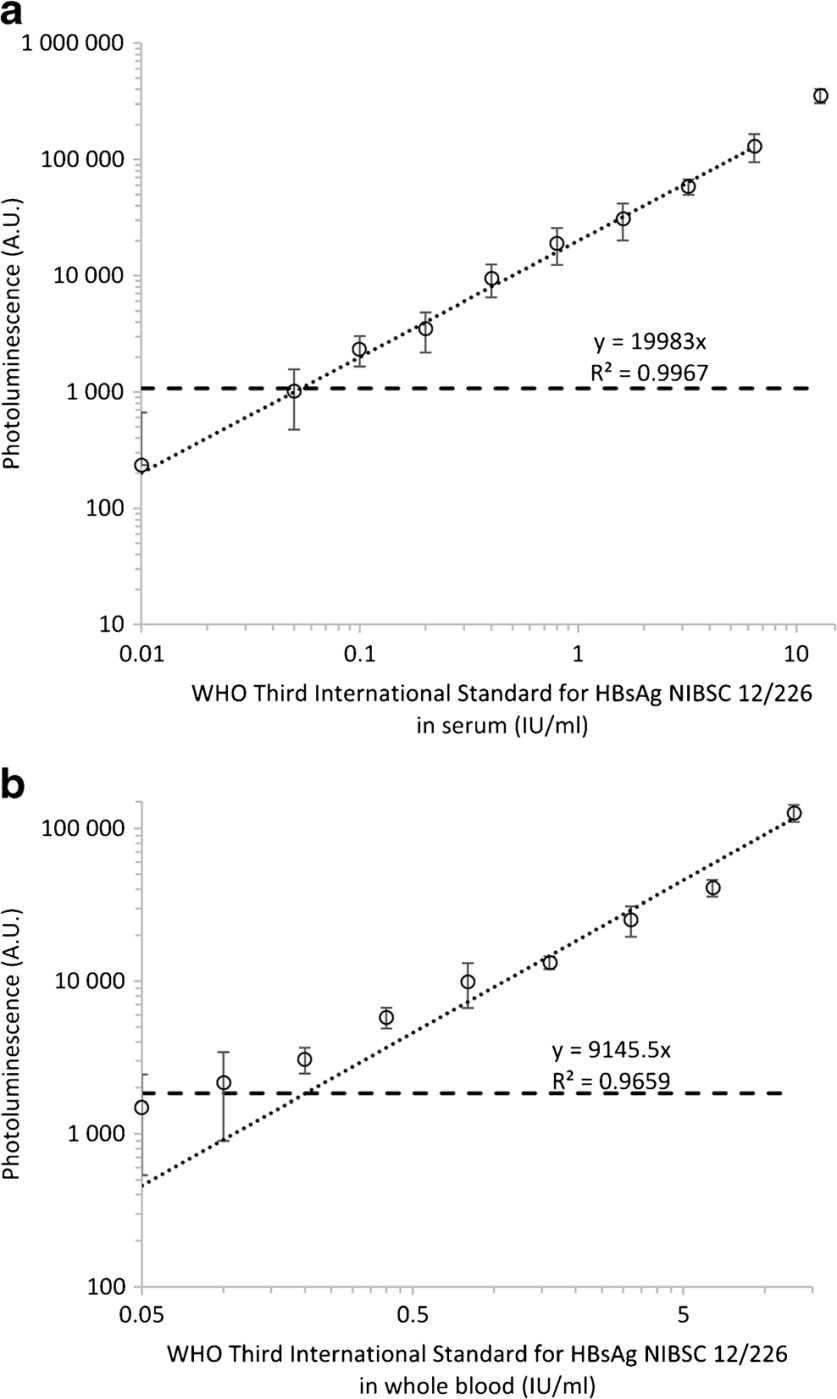
Limit of detection of the developed LFIA in **A** human serum and **B** human whole blood by using WHO third international standard for HbsAg (NIBSC 12/226). The cutoff level is shown with the dashed line. Based on the curve, the LoD in serum was 0.05 HBsAg IU/ml and in whole blood 0.2 HBsAg IU/ml

The LoD of the developed LFIA was determined in both serum and whole blood with WHO Third International Standard for HBsAg. The initial LoD calculated against the standard curve generated using serial dilution of international standard was 0.05 IU/ml in serum (Fig. 2A). Similarly, the initial LoD in whole blood was 0.2 IU/ml from the standard curve (Fig. 2B). The LoD was further validated by testing additional replicates with HBsAg concentrations close to the detection limit. With the HBsAg concentration of 0.05 and 0.1 IU/ml in serum, the assay correctly detected 61 out of 80 (76.3%) and 79 out of 80 (98.8%) replicate strips, respectively (Table 1). This suggests that the correct LoD in serum for UCNP-LFIA should be 0.1 IU/ml. In the whole blood samples, only 70 out of 80 replicates (87.5%) scored positive at a concentration of 0.2 IU/ml (Table 1). The UCNP-LFIA strip profiles from LoD measurements are shown in ESM (Fig. S11). For the comparison, the LoD of the commercial Alere Determine HBsAg LFIA was also determined in serum and found to be 3.2 IU/ml (Table 1).

**Table 1:** Verification of the limit of detection of the developed LFIA by using WHO third international standard for HBsAg (NIBSC 12/226)

WHO Third International Standard for HBsAg, NIBSC 12/226 (IU/ml)	UCNP-LFIA		Alere Determine HBsAg
	Diluted in serum (%)	Diluted in whole blood (%)	Diluted in serum
0.01	0/4 (0)	0/4 (0)	
0.05	61/80 (76.3)	3/20 (15.0)	
0.1	79/80 (98.8)	9/20 (45.0)	
0.2	20/20 (100)	70/80 (87.5)	
0.4	4/4 (100)	4/4 (100)	
0.8	4/4 (100)	4/4 (100)	0/4
1.6	4/4 (100)	4/4 (100)	0/4
3.2	4/4 (100)	4/4 (100)	4/4
6.4	4/4 (100)	4/4 (100)	4/4
12.8	4/4 (100)	4/4 (100)	2/2

The obtained analytical sensitivity of 3.2 IU HBsAg/mL for the Alere Determine HBsAg test is similar to the LoD reported (2 IU/mL) in the literature for this test [26]. Currently, poor analytical sensitivity is considered one of the key challenges in using RDTs in screening HBV infections [27]. According to the study by Scheiblauer et al., the detection limits of commercial HBsAg RDTs vary between 1.5–10 IU/ml [8]. According to the review by Khuroo et al. multiple tests had detection limit of 4 IU/ml [28]. Others have tried to increase the sensitivity of HBsAg LFIA by the use of a signal amplification system where a dual tracer is used [26], use of fluorescent nanoparticles [29], use of chemiluminescence with CdS nanowire (NW) photosensor [30], and use of self-assembled colloidal gold superparticles [31] and achieved LoDs of 0.5 IU/mL, 2.5 IU/mL, 0.5 ng/mL and 0.46 ng/mL, respectively.

The analytical sensitivity obtained by us with the developed UCNP-LFIA was 15- to 100-fold higher than the typical RDTs for HBsAg. The UCNP-LFIA showed 32-fold higher analytical sensitivity compared with the Alere Determine HBsAg LFIA that was tested in parallel. The developed UCNP-LFIA would be suitable for diagnostic purposes in terms of the analytical sensitivity requirement of ≤ 4 IU/ml [9]. Moreover, based on the detection limit obtained in serum, the UCNP-LFIA may be suitable for screening of blood products according to the WHO criteria in terms of the analytical sensitivity requirement of ≤ 0.13 IU/ml [9].

### Evaluation of UCNP-LFIA using HBsAg-positive and HBsAg-negative samples

**Fig. 3:**
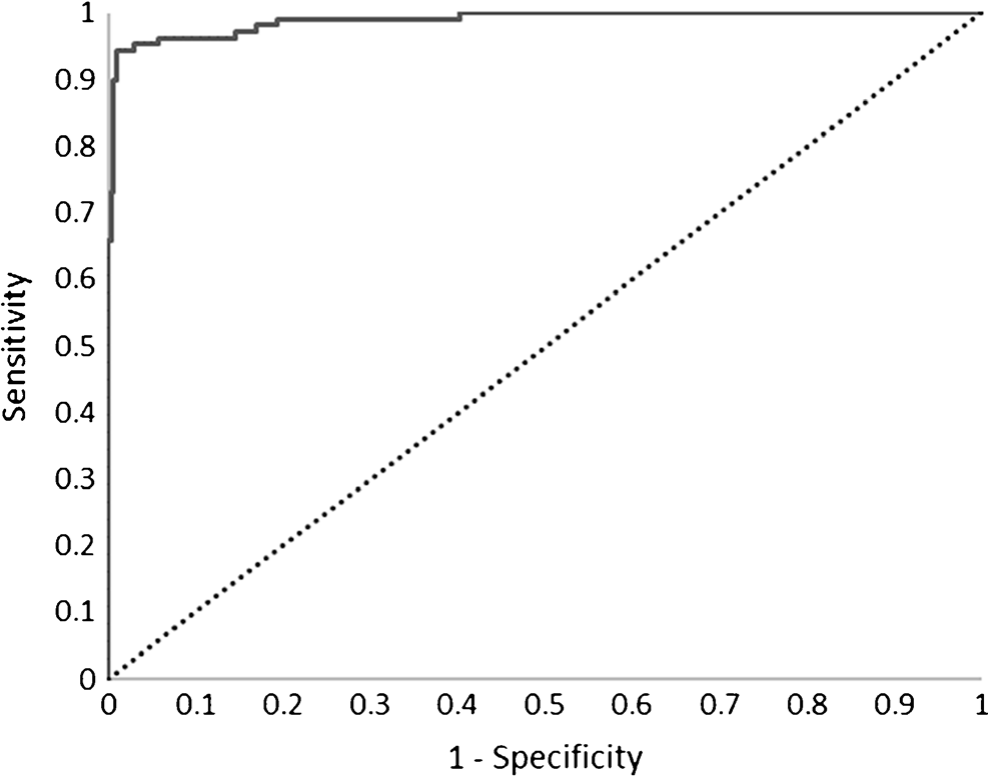
Diagnostic accuracy of UCNP-LFIA. Receiver operating characteristic (ROC) of the UCNP-LFIA was calculated based on 108 HBsAg positive and 315 HBsAg-negative samples. The area under the curve (AUC) was 0.989

A large panel of clinical samples (108 positive and 315 negative for HBsAg) characterized on central lab tests, i.e., ARCHITECT HBsAg, Bio-Rad Genetic Systems EIA, DiaSorin EIA, and bioMerieux Vidas HBsAg (ESM Table S1), were used for the determination of sensitivity and specificity of UCNP-LFIA. The ROC curve of the LFIA with the clinical samples is shown in Fig. 3. The AUC was 0.989. The sensitivity of UCNP-LFIA was 95.4% (95%CI 89.5–98.5%) based on correct determination of 103/108 HBsAg-positive samples and the specificity was 97.1% (95%CI 94.7–98.7%) based on correct determination of 306/315 negative samples. The same set of samples were tested with a commercial RDT, which resulted in a sensitivity of 87.7% (95%CI 79.9–93.3%) based on correct determination of 93/106 positive samples and specificity of 99.7% (95%CI 98.2–100%) based on correct determination of 314/315 negative samples. Two positive samples were run out and thus omitted from testing on commercial RDT. The performance comparison between UCNP-LFIA and commercial RDT is presented in Table 2.

**Table 2:** Assay performances with clinical patient samples compared between the developed LFIA and Alere Determine HBsAg™

	HBsAg LFIA	Alere Determine HBsAg™
Number of positive samples tested	108	106
Number of negative samples tested	315	315
Number of samples with agreeing result (with central lab tests)		
True positive	103	93
True negative	306	314
Number of samples with disagreeing result (with central lab tests)		
False positive	9	1
False negative	5	13
Sensitivity	95.4% (95%CI 89.5–98.5%)	87.7% (95%CI 79.9–93.3%)
Specificity	97.1% (95%CI 94.7–98.7%)	99.7% (95%CI 98.2–100%)
Total number of tested samples	423	421

### Evaluation of UCNP-LFIA using performance panels and seroconversion panel

The UCNP-LFIA performance was investigated using commercial performance panels containing extensively characterized samples scoring positive or negative on several central laboratory tests (ESM Table S1). In parallel, the commercial rapid test was evaluated using these panels. The results of evaluation using AccuSet™ HBsAg Performance Panel and AccuSet™ HBsAg Mixed Titer Performance Panel are shown in Tables 3 and 4, respectively. The UCNP-LFIA detected 20 out of 24 HBsAg-positive samples within the AccuSet™ HBsAg Performance Panel (Table 3), whereas conventional RDT detected only 8 out of 24 samples. The UCNP-LFIA recognized 8 out of 10 positive samples from the Mixed Titer Performance Panel (Table 4). In contrast, the conventional RDT could only detect 4 out of 10 positive samples in the Mixed Titer panel. We have also used a seroconversion panel to study the performance of the developed test in early infection cases. In the seroconversion panel (Table 5), samples are collected from a single donor at different time points in the early stage of HBV infection. The developed UCNP-LFIA detected HBsAg seroconversion at day 14, which was earlier than the commercial rapid test (Table 5).

**Table 3:**
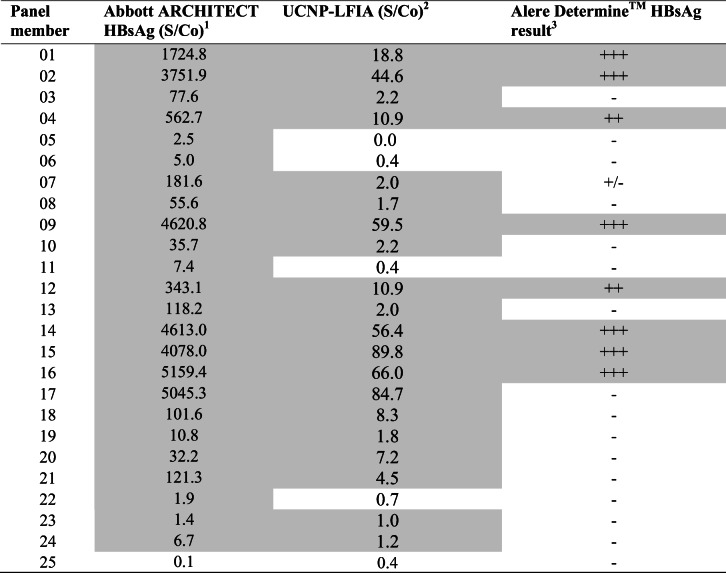
Evaluation of the developed UCNP-LFIA with AccuSet HBsAg Performance Panel and comparison with commercial RDT

Positive results are presented in gray background

^1^Results from the panel data sheet. S/Co ratios ≥1.0 are reactive

^2^For UCNP-LFIA, S/Co ratios ≥ 1.0 are considered reactive

^3^Interpretation: +++ strong visible test line; ++ visible test line; + faint visible test line; ± equivocal, − no visible test line

**Table 4:**
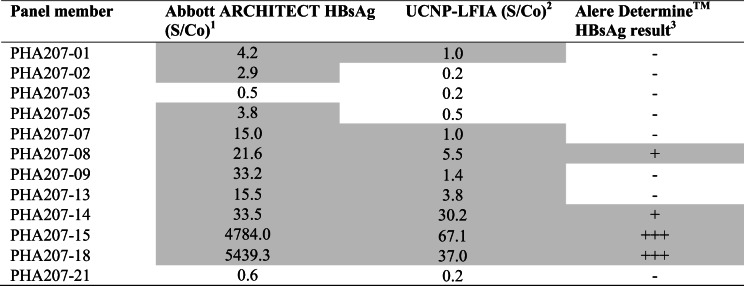
Evaluation of the developed UCNP-LFIA with AccuSet HBsAg Mixed Titer Performance Panel and comparison with Alere Determine™ HBsAg

Positive results are presented in gray background

^1^Results from the panel data sheet. S/Co ratios ≥ 1.0 are reactive

^2^For UCNP-LFIA, S/Co ratios ≥ 1.0 are considered reactive

^3^Interpretation: +++ strong visible test line; ++ visible test line; + faint visible test line; ± equivocal, − no visible test line

**Table 5:**
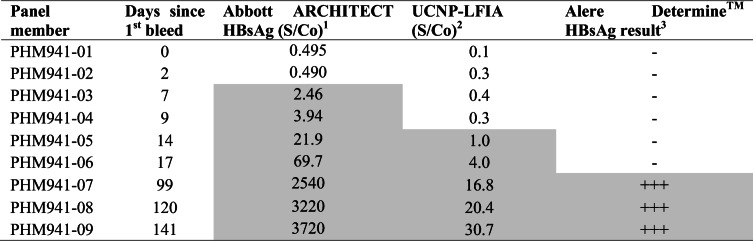
Evaluation of the developed UCNP-LFIA with HBV Seroconversion Panel and comparison with Alere Determine™ HBsAg

Positive results are presented in gray background

^1^Results from the panel data sheet. S/Co ratios ≥ 1.0 are reactive

^2^For UCNP-LFIA, S/Co ratios ≥ 1.0 are considered reactive

^3^Interpretation: +++ strong visible test line; ++ visible test line; + faint visible test line; ± equivocal, − no visible test line

### Evaluation of UCNP-LFIA using HBV genotype panels

There is a concern that genetic variants of HBV affect the sensitivities of HBsAg assays or even escape the detection from assays relying on monoclonal antibodies only [32–34]. Genetic variation in HBsAg has been demonstrated to affect the sensitivities of HBsAg detection assays [35] and therefore there is a need to use pairs of antibodies that detect HBsAg from all major HBV genotypes/variants. In the UCNP-LFIA, three non-competing monoclonal capture antibodies were applied alongside the monoclonal tracer antibody that was selected based on the binding with the major HBV genotypes (see ESM, Figs. S4 and S5 and Tables S2 and S3). The detection of HBV genotype variants by the developed LFIA was ensured by evaluating the assay with the 1st WHO international reference panel for HBV genotypes for HBsAg assays (6100/09) and the AccuSet™ HBV worldwide performance panel. The results of the genotype detection are shown in Table 6. The UCNP- LFIA as well as the commercial RDT were able to detect all the HBV genotypes.

**Table 6:**
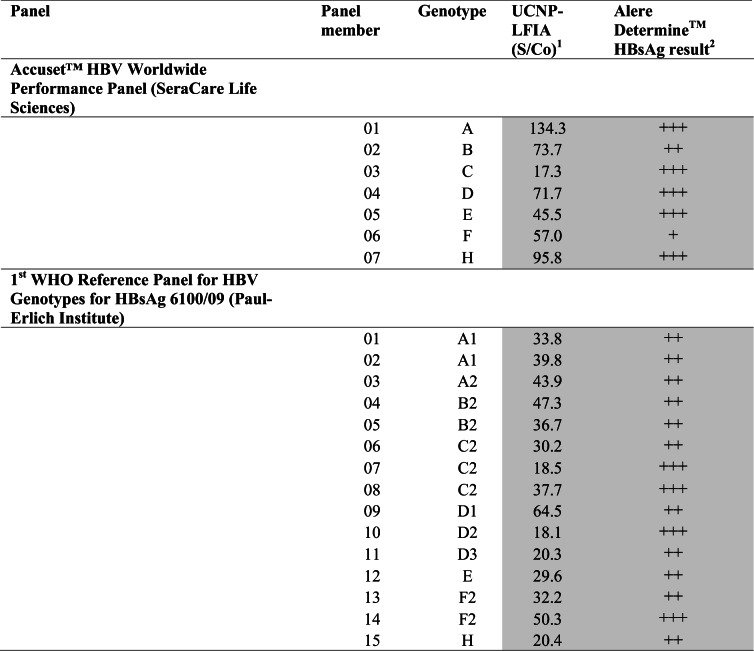
Evaluation of the developed UCNP-LFIA with HBV genotype panels

Positive results are presented in gray background

^1^S/Co ratios ≥1.0 are considered reactive

^2^Interpretation: +++ strong visible test line; ++ visible test line; + faint visible test line; ± equivocal, − no visible test line

### Stability of UCNP-LFIA strips

**Fig. 4:**
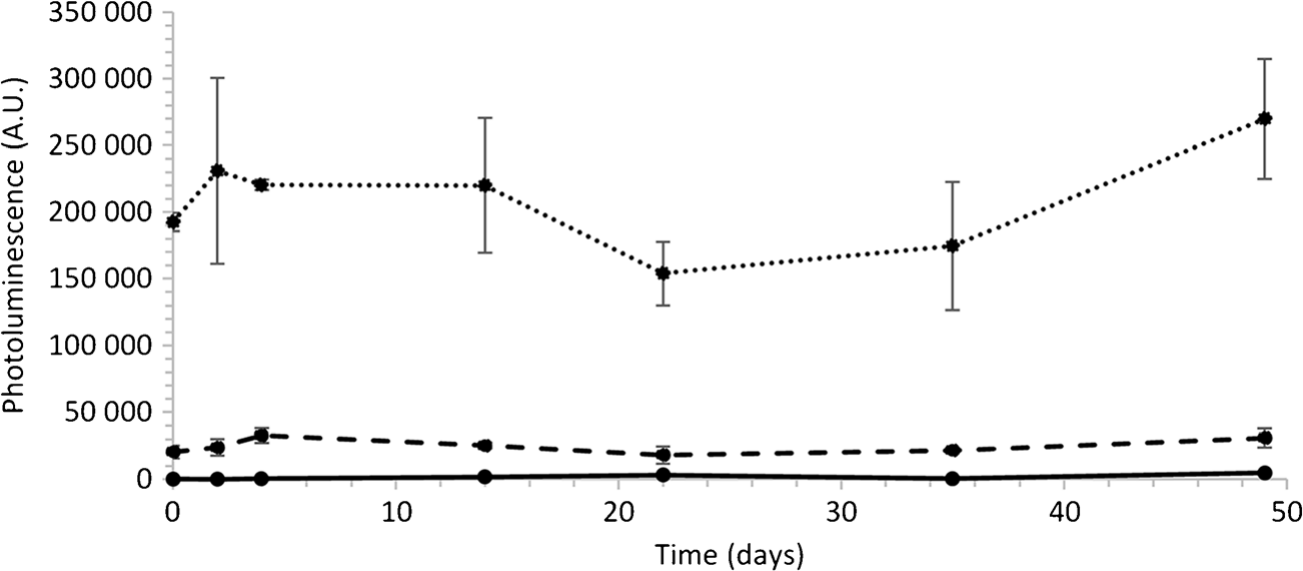
UCNP-LFIA stability at 37 °C. The stability of the strips was tested at 7 time points during 50 days with HBsAg concentrations 0 ng/ml (solid line), 0.5 ng/ml (dashed line), and 5 ng/ml (dotted line) in serum. Error bars represent the standard deviation of 3 replicates

A stability study of the UCNP-LFIA strips was performed at 37 °C for 50 days. The strips were tested at 7 time points during the study period. The data suggest that the test is stable with no significant change in the performance even for the lower concentration of HBsAg tested (Fig. 4). The LFIA strips generally have a good shelf-life and are often stored at room temperature. The endurance in varying conditions, which may occur during transportation and storage, allows the delivery of the test to the end-users in low-resource settings [36].

### Photoluminescence measurement reproducibility

As the UCNP-LFIA requires a reader device to read the strip, it is essential to analyze the reproducibility in the photoluminescence measurement. For this purpose, we have performed 15 repeat measurements of the same strips with varying amounts of analytes and found coefficients of variation (CV) of 2.8%, 2.0%, and 1.8% for strips run with 0, 0.5, and 5 ng/ml HBsAg, respectively (ESM Fig. S9). This result suggests that the reader device can provide consistent photoluminescence readout. We have also compared the performance of a benchtop UCNP reader with a battery-operated portable UCNP reader and found similar sensitivity for both the reader for measuring signal from UCNPs on the strip (ESM Fig. S10).

### Correlation between UCNP-LFIA and Architect S/co ratios

**Fig. 5:**
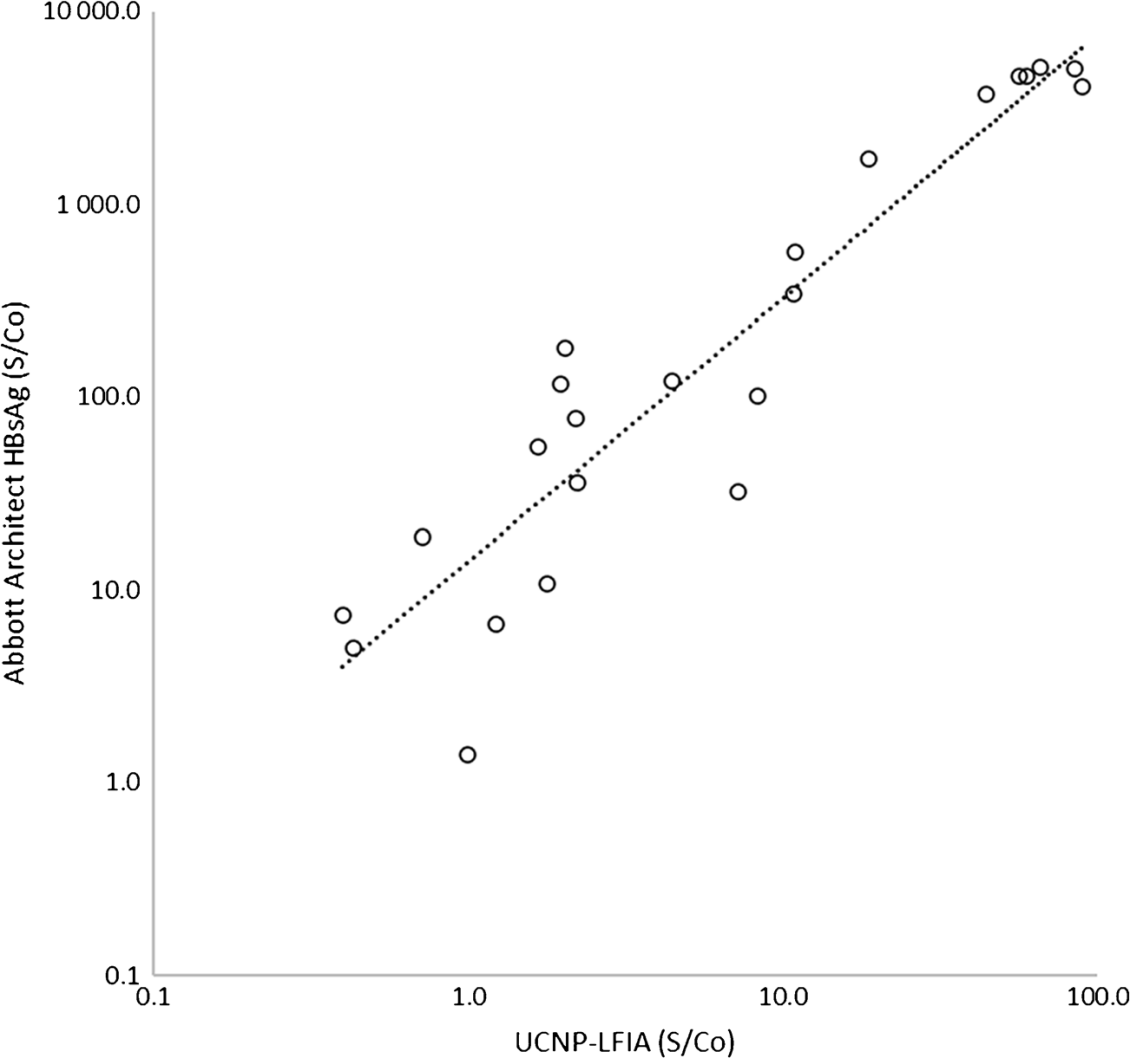
Correlation of UCNP-LFIA and Abbott Architect HBsAg signal-to-cutoff ratios (S/Co) with the AccuSet HBsAg Performance Panel samples. *R*^2^ = 0.87

As the UCNP technology enables the analyte quantification in LFIA format [14, 16], correlation in S/Co ratios of the developed UCNP-LFIA and Abbott Architect (automated chemiluminescent immunoassay) was examined, and it was found to be reasonable (*R*^2^ = 0.87) (Fig. 5). Quantitative measurement of HBsAg can be used for guiding therapeutic indications [37], particularly in LMICs where methods to detect and quantify HBV DNA are often unavailable. As UCNP technology uses a reader device and results are quantifiable, the developed UCNP-LFIA may also find its utility in semi-quantitative determination of HBsAg concentration.

## Conclusions

In LMICs, RDTs are often used instead of EIAs as RDTs are low cost and do not require much infrastructure and trained personnel. Due to this, there is a need for highly sensitive RDTs detecting HBsAg, particularly for the screening of donated blood [3]. In this work, we developed a UCNP-LFIA, which is more sensitive than the commercial visually read RDT. In the future, the assay will be evaluated in clinical settings, with freshly drawn patient samples, using a battery-operated reader device feasible for point-of-care use to evaluate the true performance of the UCNP-LFIA. High-sensitivity rapid tests for HBsAg are necessary with the effective vaccination program to eliminate Hepatitis B.

## Supplementary Information

ESM 1 (PDF 666 kb).

## Data Availability

Not applicable.
