# Supplementary material for: Upconverting nanoparticle reporter–based highly sensitive rapid lateral flow immunoassay for hepatitis B virus surface antigen
Source: Anal Bioanal Chem. 2020 Nov 23;413(4):967–78. doi: 10.1007/s00216-020-03055-z (PMC7813740; doi:10.1007/s00216-020-03055-z)
Supplement: Supplementary file 1 — (PDF 666 kb). [file 216_2020_3055_MOESM1_ESM.pdf]

**Analytical and Bioanalytical Chemistry**

**Electronic Supplementary Material**

# Upconverting nanoparticle reporter based highly sensitive rapid lateral flow immunoassay for hepatitis B virus surface antigen

Iida Martiskainen, Sheikh M. Talha, Karoliina Vuorenpää, Teppo Salminen, Etvi Juntunen, Souvick Chattopadhyay, Dinesh Kumar, Tytti Vuorinen, Kim Pettersson, Navin Khanna, Gaurav Batra

## CONTENTS

Details about Reference Assays
**Table S1.** Samples used in UCNP-LFIA evaluation.
**Fig. S1.** Reactivity of hybridoma culture supernatants with HBsAg.
**Fig. S2.** Screening of tracer antibody.
**Fig. S3.** Competitive binding inhibition assay.
**Table S2.** Inhibition percentages resulted from the competitive binding inhibition assay.
**Fig. S4.** Reactivity of different capture antibodies to genotype panel members.
**Fig. S5.** Grouping of mAbs based on competitive binding inhibition assay and genotype detection.
**Table S3.** The analytical sensitivities obtained with different capture mAbs combinations.
**Fig. S6.** The effect of the UCNP-mAb conjugate drying.
**Table S4.** Nitrocellulose membranes used for the comparison.
**Fig. S7.** Nitrocellulose membrane selection.
**Fig. S8.** Effect of whole blood volume on test performance.
**Fig. S9.** UCNP photoluminescence measurement reproducibility.
**Fig. S10.** Comparison of reader devices.
**Fig. S11.** UCNP-LFIA strip profiles from the LoD determination experiment.

**Reference Assays**

The status of the sample as HBsAg positive was based on results in the central laboratory assays as listed in Table S1. The ARCHITECT HBsAg assay (Abbott Laboratories, USA) is an automated chemiluminescent microparticle immunoassay for the detection of HBsAg in human serum or plasma. The LIAISON® XL MUREX HBsAg assay (DiaSorin Inc.) is an automated chemiluminescence immunoassay platform for the detection of HBsAg in human serum or plasma samples. VIDAS HBsAg (BioMérieux, France) is an automated enzyme linked fluorescent assay for the detection of HBsAg in human serum or plasma samples. Genetic Systems HBsAg assay (Bio-Rad Laboratories Inc., USA) is a manual (compatible with automation) sandwich ELISA test for the detection of HBsAg in human serum or plasma samples.

**Table S1** Samples used in UCNP-LFIA evaluation

| Sample/sample panel | Purchased from | Number of Samples | Sample matrix | Reference assay |
|---|---|---|---|---|
| WHO Third International Standard for HBsAg (12/226) | National Institute for Biological Standards and Control, UK | 1 | plasma | NA |
| The 1st WHO International Reference Panel for Hepatitis B Virus (HBV) Genotypes for Hepatitis B Surface Antigen (HBsAg) Assays (6100/09) | Paul-Ehrlich Institut, Germany | 15 | plasma | NA |
| AccuSet™ HBsAg Performance Panel (0805-0340) | SeraCare Life Sciences Inc., USA | 25 | plasma | ARCHITECT HBsAg |
| AccuSet™ HBsAg Mixed Titer Performance Panel Modified PHA207(M) (0805-0217) | SeraCare Life Sciences Inc., USA | 12 | plasma | ARCHITECT HBsAg |
| AccuSet™ HBV Worldwide Performance Panel (0805-0313) | SeraCare Life Sciences Inc., USA | 7 | plasma | ARCHITECT HBsAg |
| HBV Seroconversion Panel PHM941 (0606-0060) | SeraCare Life Sciences Inc., USA | 9 | plasma | ARCHITECT HBsAg |
| HBsAg positive disease state samples | SeraCare Life Sciences Inc., USA | 24 | plasma | Abbott EIA/Bio-Rad Genetic Systems EIA/DiaSorin EIA |
| HBsAg positive disease state samples | Biomex GmbH, Germany | 49 | serum | ARCHITECT HBsAg |
| HBsAg positive disease state samples | Labquality Oy, Finland | 18 | plasma | ARCHITECT HBsAg and bioMerieux Vidas HBsAg |
| Presumed healthy samples | Turku University of Applied Sciences, Finland | 100 | serum | Alere Determine HBsAg |
| Presumed healthy samples | Turku University of Applied Sciences, Finland | 1 | whole blood | Alere Determine HBsAg |
| | **Obtained from** | | | |
| Routine-tested clinical samples negative for HBsAg | Department of Virology, University of Turku, Finland | 215 | plasma, serum | ARCHITECT HBsAg Qualitative immunoassay |
| Routine-tested clinical samples positive for HBsAg | Department of Virology, University of Turku, Finland | 16 | plasma, serum | ARCHITECT HBsAg Qualitative immunoassay |

NA = information not available

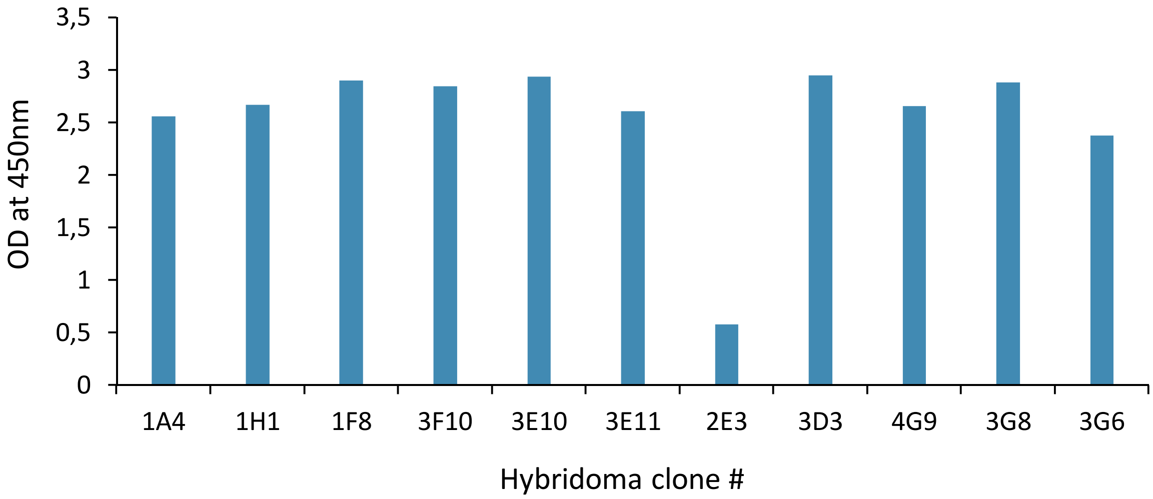


**Fig. S1** Reactivity of hybridoma culture supernatants with HBsAg. The reactivity of mouse anti-HBsAg antibodies, from hybridoma culture supernatants, were determined using indirect ELISA method. X-axis represents the clone # and the y-axis represents the OD at 450nm

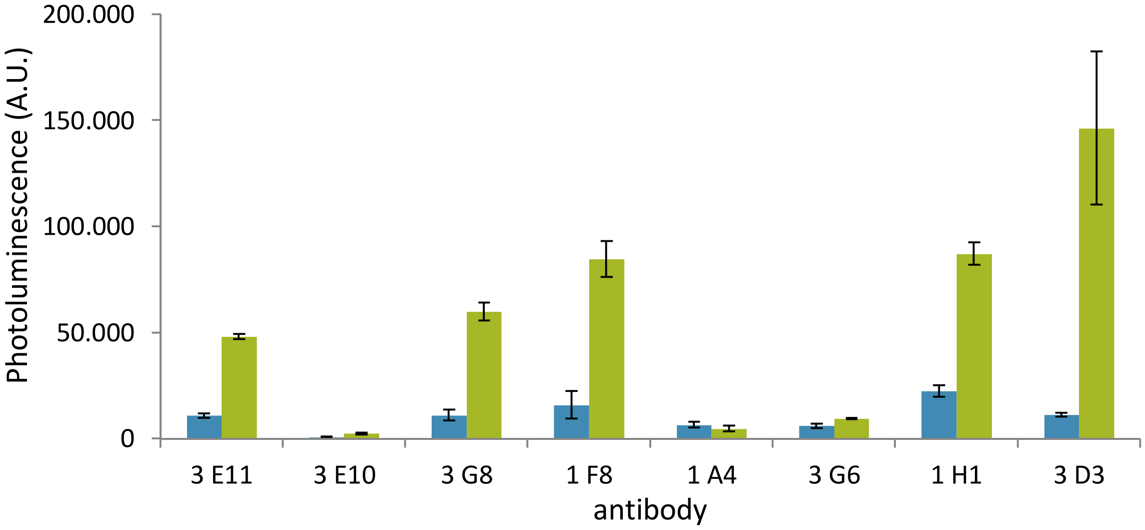


**Fig. S2** Screening of tracer antibody. Eight mAbs were conjugated to the UCNP surface and screened in a dipstick format. Goat polyclonal anti-HBsAg antibody was used as a capture. The produced mAb-UCNP-conjugates were initially screened using blank goat serum (blue) and 100 ng/ml native HBsAg spiked in goat serum (green). The mAb-3D3 was observed to result in the highest specific photoluminescence signal and the highest signal-to-background ratio. In the strip profiles of the lateral flow strips, 3D3-UCNP-conjugate had the best flow properties on the nitrocellulose, and no photoluminescence signal at the nitrocellulose entry point (indicating that the non-specific retention of UCNPs to be minimal). X-axis represents the mAb clones and y-axis represents the test line signal from the strip

$$\text{Inhibition \%} = 100 - \frac{\text{specific signal}}{\text{specific signal (control reaction)}} \times 100$$

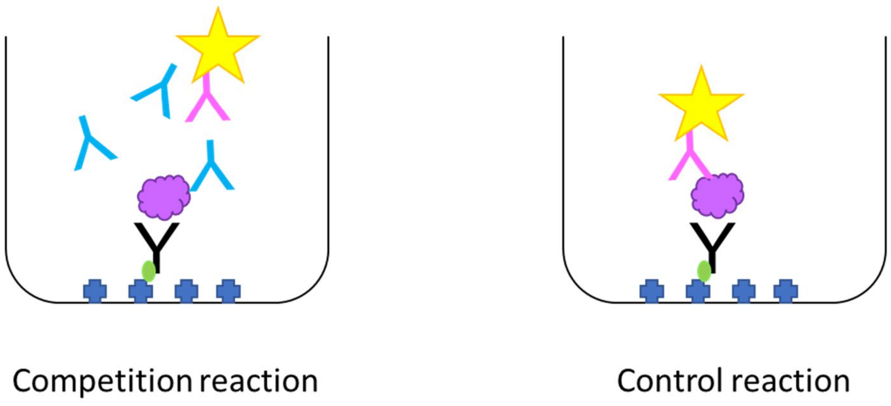


**Fig. S3** Competitive binding inhibition assay. The mAbs were tested against each other both as free inhibitors and Eu(III)-chelate labeled tracers to investigate whether they bind to the same epitope on HBsAg. Eu(III)-chelate-labeled mAb was allowed to compete with an excess of free mAb in the reaction with HBsAg. Biotinylated anti-HBsAg goat polyclonal antibody was used as capture on streptavidin coated wells. The amount of competing mAb was optimized to ensure a sufficient level of inhibition prior to performing the competitive binding inhibition assay. The inhibition percentage was calculated by subtracting the signal obtained from the competitive reaction from the signal from control reaction (no competing mAb present) and dividing by the control reaction signal. In case of high inhibition percentage, it was assumed that the competing antibody binds to overlapping epitope as the Eu(III)-labeled tracer-mAb, whereas with low inhibition percentage it was assumed that the competing antibody binds to non-overlapping epitope

**Table S2** Inhibition percentages resulted from the competitive binding inhibition assay

| | **Tracer mAb** | | | | | | | | |
|---|---|---|---|---|---|---|---|---|---|
| **Inhibiting mAb**[a] | *2505* | *1H1* | *3G8* | *1F8* | *3F10* | *3E10* | *4G9* | *3G6* | *3D3* |
| *2505*[b] | 97.3 | 83.9 | 18.3 | 6.3 | 81.5 | 16.1 | 81.6 | 82.4 | 18.2 |
| *2508*[b] | 96.0 | 96.7 | 25.1 | 16.9 | 96.5 | 29.8 | 96.6 | 93.7 | 29.9 |
| *1H1* | 19.9 | 94.7 | 0.7 | 0.0 | 95.5 | 8.7 | 91.6 | 91.1 | 0.0 |
| *3G8* | 0.0 | 20.8 | 95.1 | 91.8 | 8.7 | 94.8 | 5.6 | 37.6 | 93.0 |
| *1F8* | 3.2 | 24.3 | 97.8 | 96.3 | 9.0 | 97.8 | 2.7 | 28.2 | 96.5 |
| *3F10* | 31.3 | 96.9 | 5.7 | 0.0 | 96.1 | 12.8 | 95.7 | 91.8 | 0.1 |
| *3E10* | 7.4 | 13.7 | 96.9 | 94.7 | 5.3 | 96.6 | 5.0 | 37.1 | 92.4 |
| *4G9* | 17.3 | 97.1 | 5.4 | 0.0 | 97.7 | 10.4 | 97.1 | 91.7 | 0.0 |
| *3G6* | 34.6 | 90.4 | 34.7 | 33.8 | 87.5 | 31.8 | 87.3 | 100.0 | 26.3 |
| *3D3* | 9.7 | 22.5 | 98.3 | 96.6 | 15.9 | 98.4 | 4.5 | 29.1 | 97.7 |

[a]Antibodies showing > 80% of inhibition (grey background) were considered to have overlapping epitope. Three different groups were obtained by using this criterion.
[b]mAbs purchased from Medix Biochemica Oy Ab, Finland

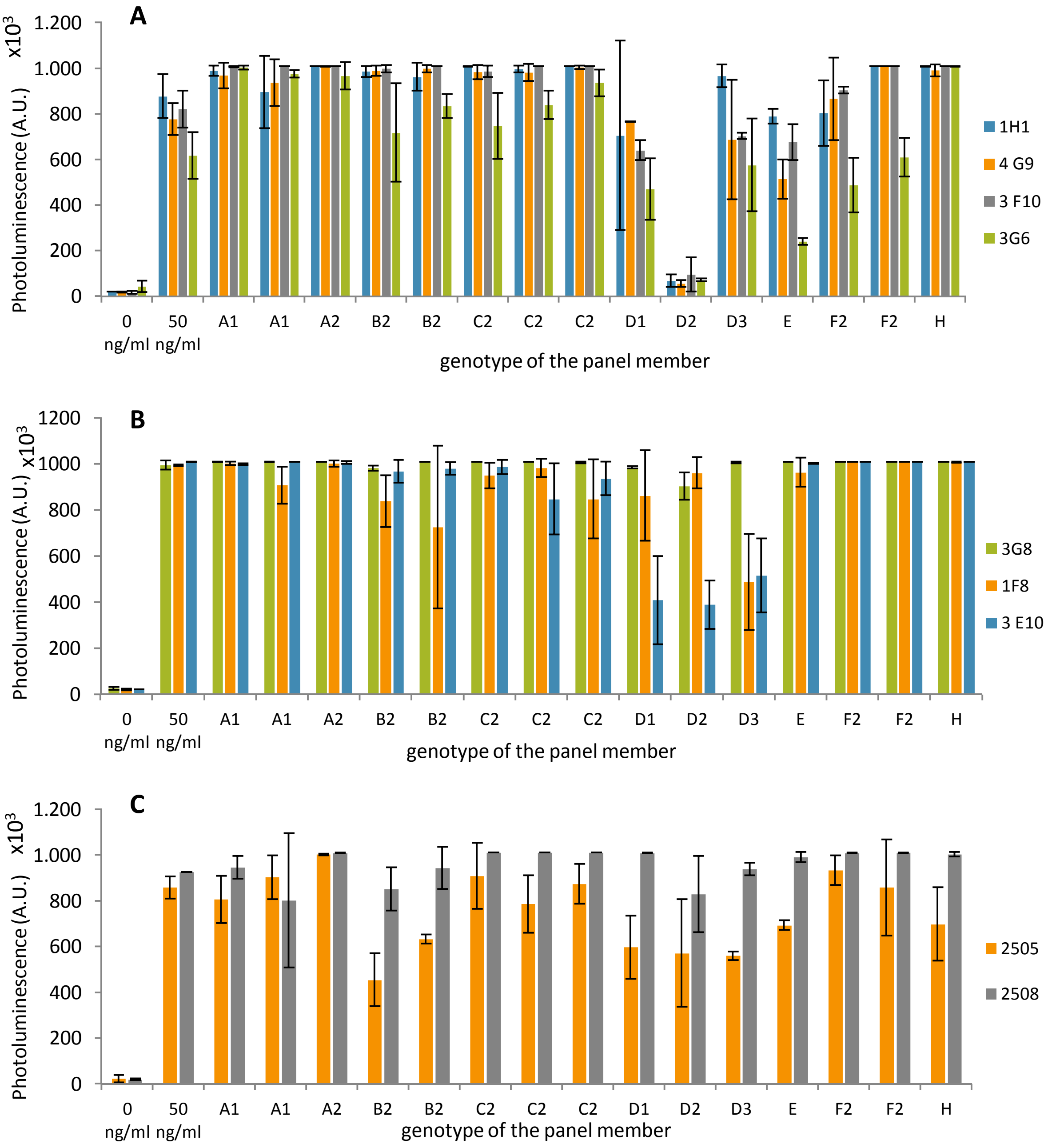


**Fig. S4** Reactivity of different capture antibodies to genotype panel members. The mAbs that worked as captures in the LFA with 3D3-UCNP-conjugate were tested with 1st WHO Reference Panel for HBV Genotypes for HBsAg (PEI 6100/09). HBsAg-spiked serum samples of 0 and 50 ng/ml were used as negative and positive control, respectively. A) 1H1 (blue), 4G9 (orange), 3F10 (grey) and 3G6 (green). B) 3G8 (green), 1F8 (orange) and 3E10 (blue). C) 2505 (orange) and 2508 (grey). MAbs 1H1, 4G9, 3F10 and 3G6 showed weaker reactivity to genotype D2

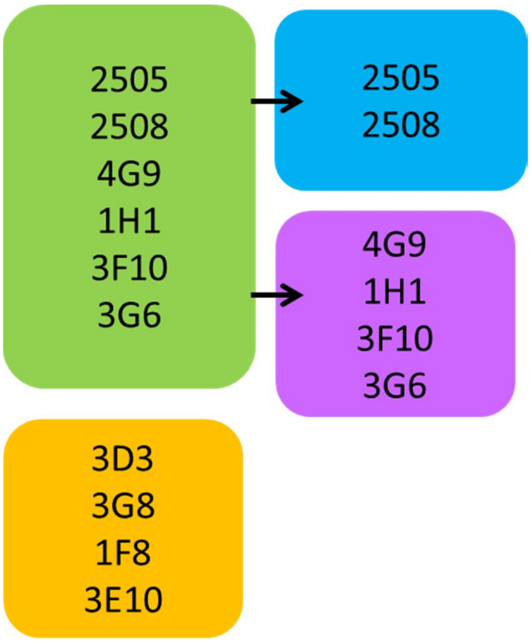


**Fig. S5** Grouping of mAbs based on competitive binding inhibition assay and genotype detection. Competitive binding inhibition assay resulted in two different groups: mAb-2505, mAb-2508, mAb-4G9, mAb-1H1, mAb-3F10 and mAb-3G6 (group 1, green box) and mAb-3D3, mAb-3G8, mAb-1F8 and mAb-3E10 (group 2, yellow box). Group 1 was further divided into two groups based on the results from evaluation of the 1st WHO reference panel (PEI): mAbs with weak reactivity to genotype D2 (4G9, 1H1, 3F10 and 3G6; presented in purple box) and strong reactivity to genotype D2 (2505 and 2508; presented in blue box). Capture antibody combinations were selected from different groups to increase the probability of targeting most HBsAg variants. Overall ability to detect multiple genotypes of mAb candidates was considered when mAbs were selected for further studies

**Table S3** The analytical sensitivities obtained with different capture mAbs combinations

| Capture combination | Sensitivity (ng/ml)[a] |
|---|---|
| **3G8** | 1.0 |
| **3G8 + 2508** | 0.5 |
| **3G8 + 2508 + 4G9**[b] | 0.4 |

[a]Preliminary analytical sensitivities of the assay were determined by using HBsAg-spiked goat serum within the concentration range of 0.05 to 100 ng/ml. The cut-off was calculated as 3*SD of the blank when specific photoluminescence signals were used. The equation was obtained with linear regression of the standard curve.

[b]The highest sensitivity was achieved using the combination of monoclonal antibodies 3G8, 4G9 and 2508 as capture.

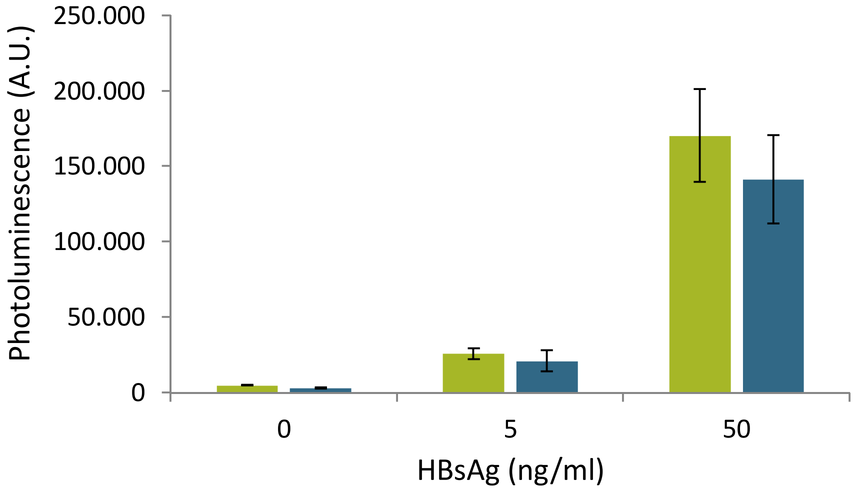


**Fig. S6** The effect of the UCNP-mAb conjugate drying. Performance of the liquid UCNP-reporters are presented with grey bars and that of dried tracer with blue bars. The mAb-3D3-UCNP-bioconjugate was dried on the conjugate pads of the LF strips with 5 % sucrose drying buffer. The effects of the transition to the dry-reagent format were compared to a dipstick assay with UCNP-reporters in solution. The assay was tested with HBsAg-spiked serum in concentrations of 0, 5 and 50 ng/ml. The drying of the reporters did not have an impact on the signal levels

**Table S4** Nitrocellulose membranes used in the comparison

| Nitrocellulose membrane | Supplier | Wicking rate (s/4cm) |
|---|---|---|
| CNPH-N SS60 | Advanced Microdevices (India) | 200 |
| CNPH-N SS40 | Advanced Microdevices (India) | 150 |
| CNPC SS12, 12 µm | Advanced Microdevices (India) | 120 |
| CNPC SS12, 10 µm | Advanced Microdevices (India) | 140 |
| CNPF SN12, 10 µm | Advanced Microdevices (India) | 125 |
| CNPF SN12, 8 µm | Advanced Microdevices (India) | 185 |
| LFNC-C-BS023_70 | Nupore Filtration Systems (India) | 69 |
| LFNC-C-SS22_70 | Nupore Filtration Systems (India) | 69 |
| LFNC-C-SS22, 15 µm | Nupore Filtration Systems (India) | 79 |
| LFNC-C-BS026, 15 µm | Nupore Filtration Systems (India) | 79 |
| LFNC-C-BS026, 10 µm | Nupore Filtration Systems (India) | 114 |
| HF75 | Millipore Corporation (USA) | 75 |
| HF90 | Millipore Corporation (USA) | 90 |
| HF120 | Millipore Corporation (USA) | 120 |

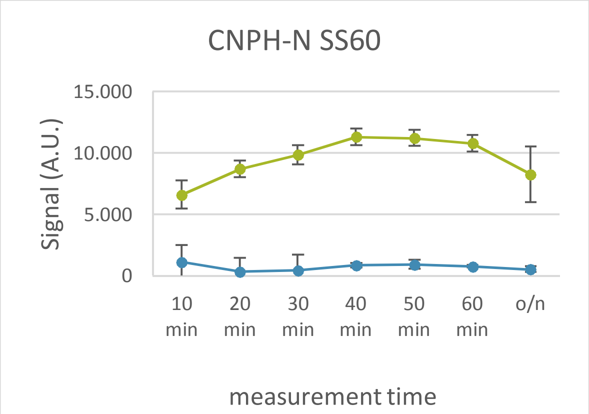
CNPH-N SS60
Signal (A.U.)
15.000
10.000
5.000
0
10 min
20 min
30 min
40 min
50 min
60 min
o/n
measurement time

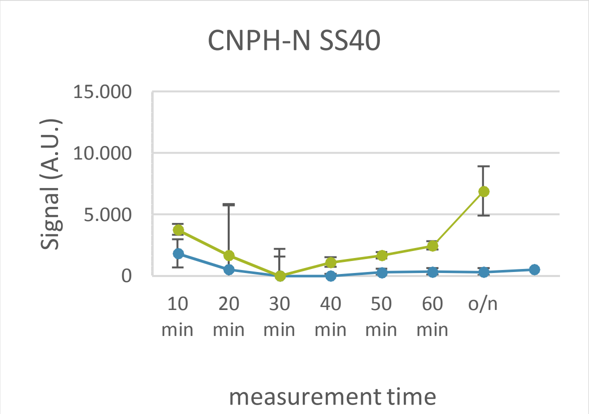
CNPH-N SS40
Signal (A.U.)
15.000
10.000
5.000
0
10 min
20 min
30 min
40 min
50 min
60 min
o/n
measurement time

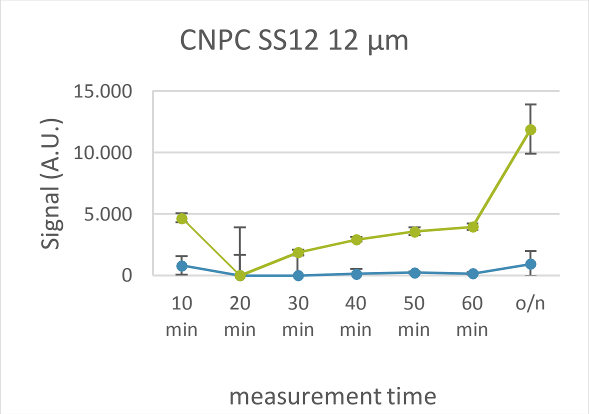
CNPC SS12 12 µm
Signal (A.U.)
15.000
10.000
5.000
0
10 min
20 min
30 min
40 min
50 min
60 min
o/n
measurement time

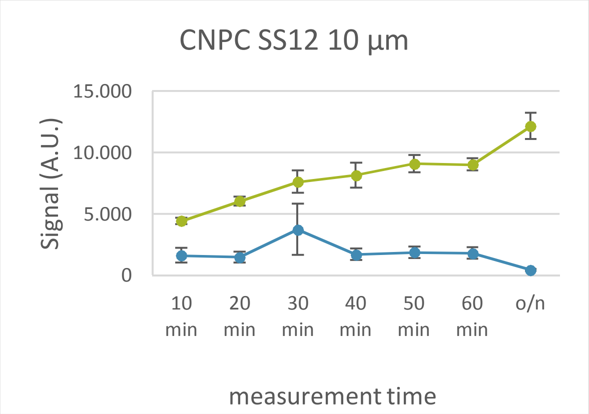
CNPC SS12 10 µm
Signal (A.U.)
15.000
10.000
5.000
0
10 min
20 min
30 min
40 min
50 min
60 min
o/n
measurement time

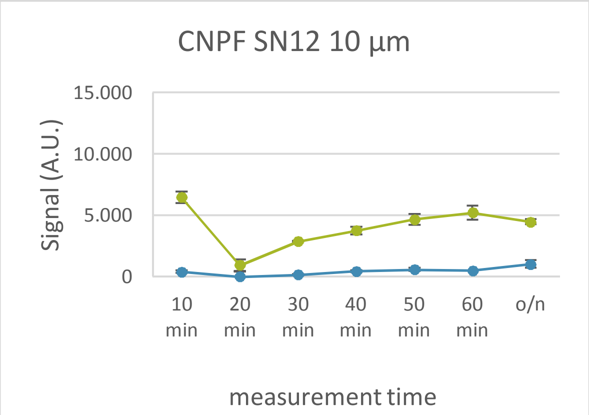
CNPF SN12 10 µm
Signal (A.U.)
15.000
10.000
5.000
0
10 min
20 min
30 min
40 min
50 min
60 min
o/n
measurement time

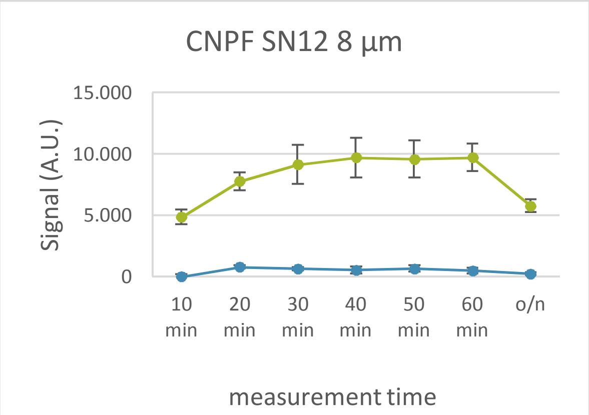
CNPF SN12 8 µm
Signal (A.U.)
15.000
10.000
5.000
0
10 min
20 min
30 min
40 min
50 min
60 min
o/n
measurement time

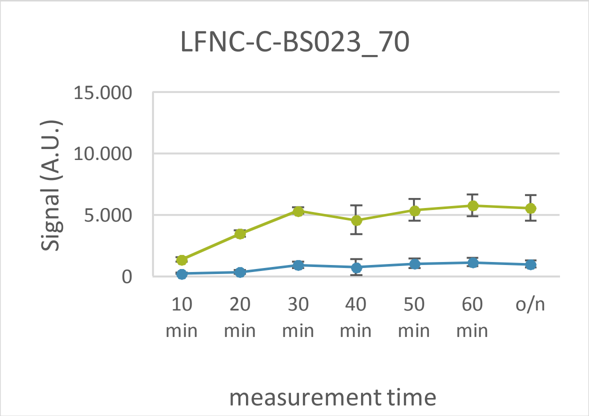
LFNC-C-BS023_70
Signal (A.U.)
15.000
10.000
5.000
0
10 min
20 min
30 min
40 min
50 min
60 min
o/n
measurement time

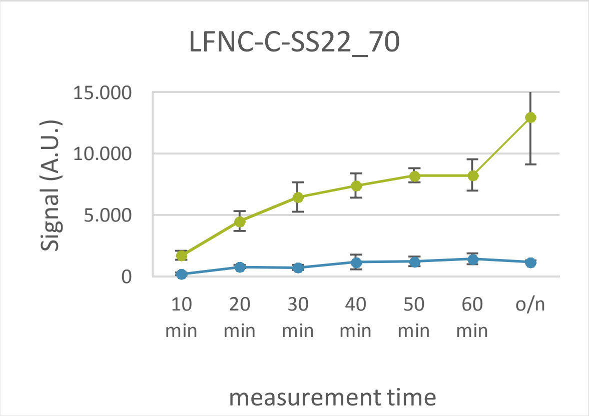
LFNC-C-SS22_70
Signal (A.U.)
15.000
10.000
5.000
0
10 min
20 min
30 min
40 min
50 min
60 min
o/n
measurement time

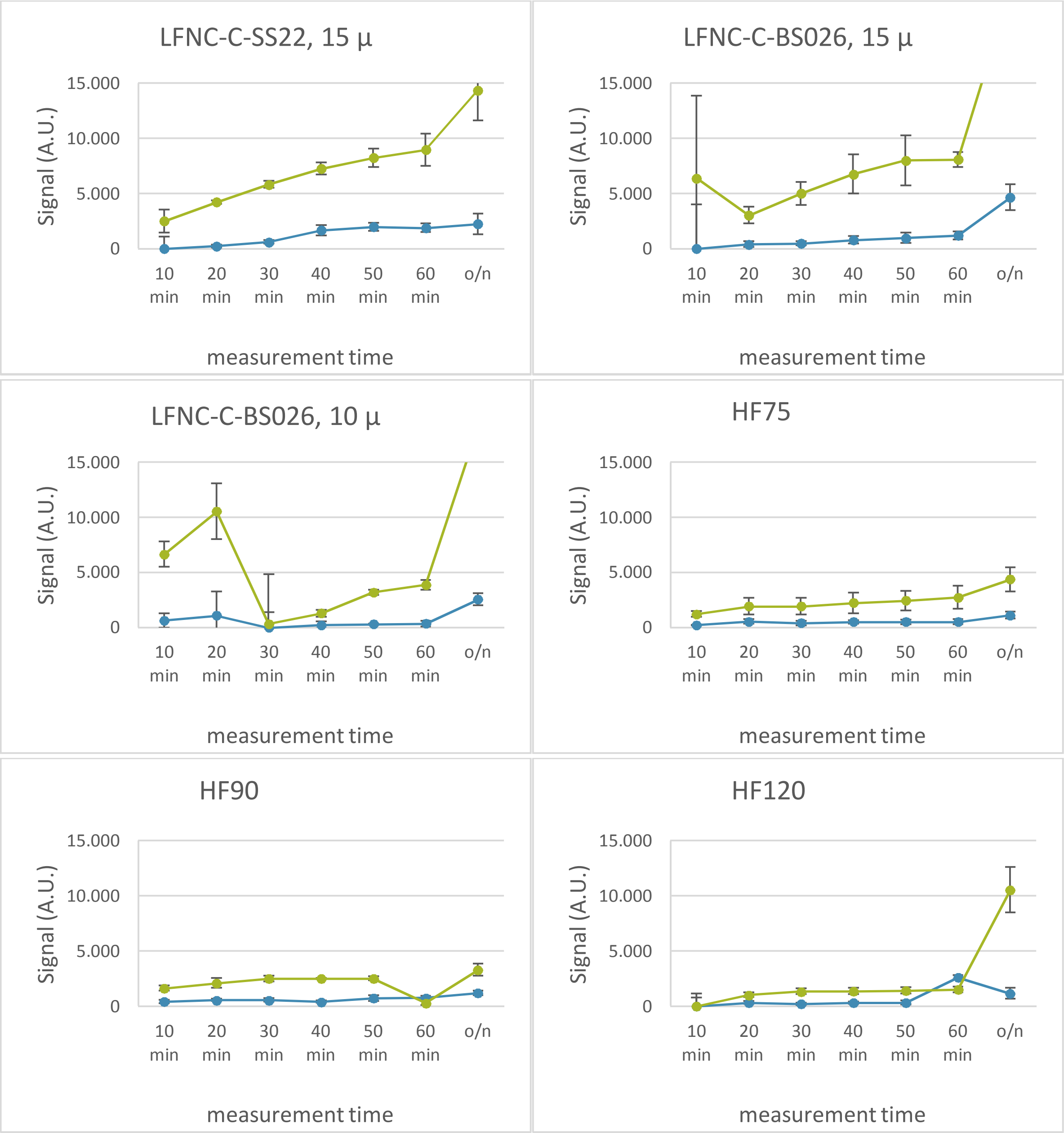


**Fig. S7** Nitrocellulose membrane selection. Fourteen different nitrocellulose membranes (NCM) were tested for UCNP-LFIA with dried UCNP conjugates. The LFIA was performed with spiked whole blood samples with HBsAg concentrations of 0 ng/ml (blue lines) and 4 ng/ml (green lines) in three replicates per concentration.The strips were measured at 10 minutes intervals and once the strips were dried overnight. Some of the NCMs tested had an initially high signal peak at the 10-minute time point, which then decreased over time. These signals were disregarded when choosing the most reliable and sensitive NCM, since the peak was probably caused by transient weak binding to the test line and was washed away at the later time-points. The chosen nitrocellulose membrane CNPH-N SS60 had a consistent signal increase over reasonable turn-around-time with maximum difference in signal intensity of positive and negative sample at the 20-minute time point. Reading at 30 minutes was considered optimal because of good combination between speed and antigen-specific signal

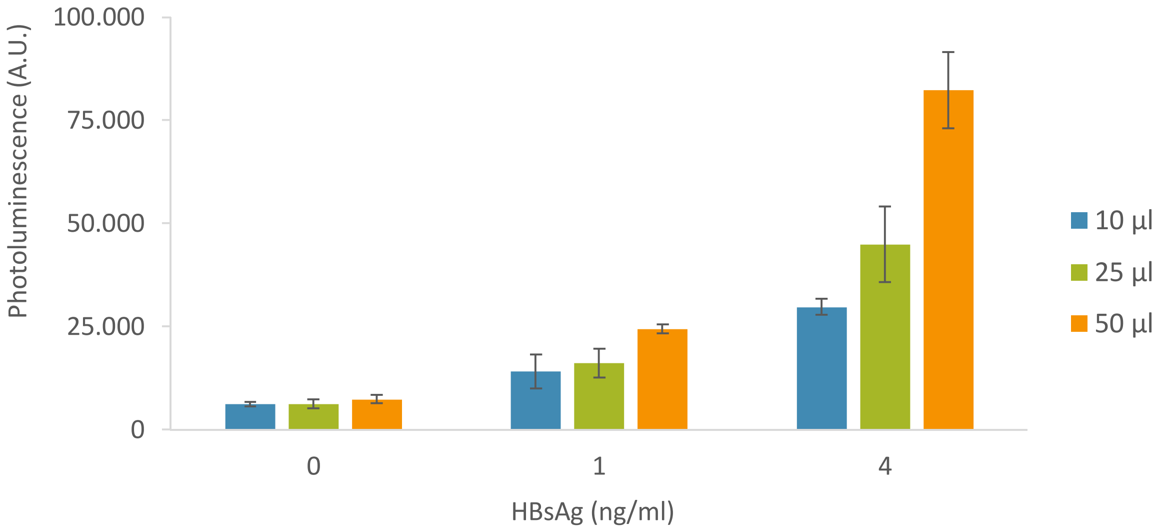


**Fig. S8** Effect of whole blood volume on test performance. Sample volume was optimised by applying either 10 µl, 25 µl or 50 µl sample and either 90 µl, 75 µl or 50 µl chase buffer, respectively. Signal levels incresed in the presence of HBsAg when the sample volume was increased, whereas the background level remained relatively low

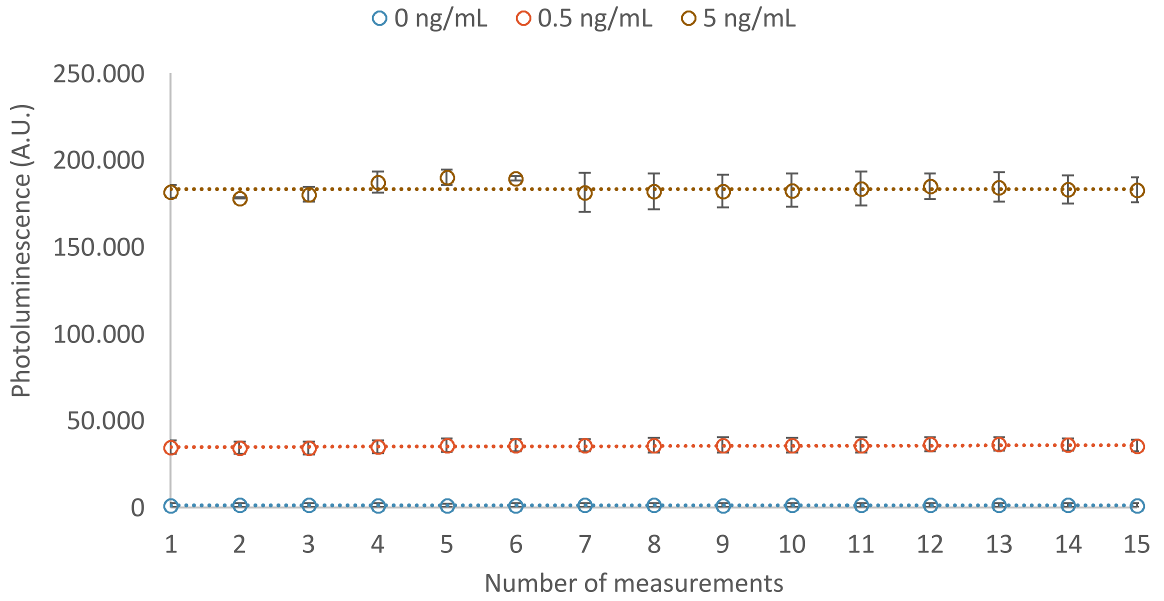


**Fig. S9** Reproducibility of the upconversion photoluminescence measurements from the UCNP-LFIA strips. Each HBsAg concentration was assayed in three replicates and each LFIA strip was measured 15 times using Labrox Upcon reader. CV% of 15 measurements for average signal measured from strips assayed with 0, 0.5 and 5 ng/mL HBsAg concentrations were 2.8%, 2.0% and 1.8, respectively. Error bars represent variation from three replicate strips

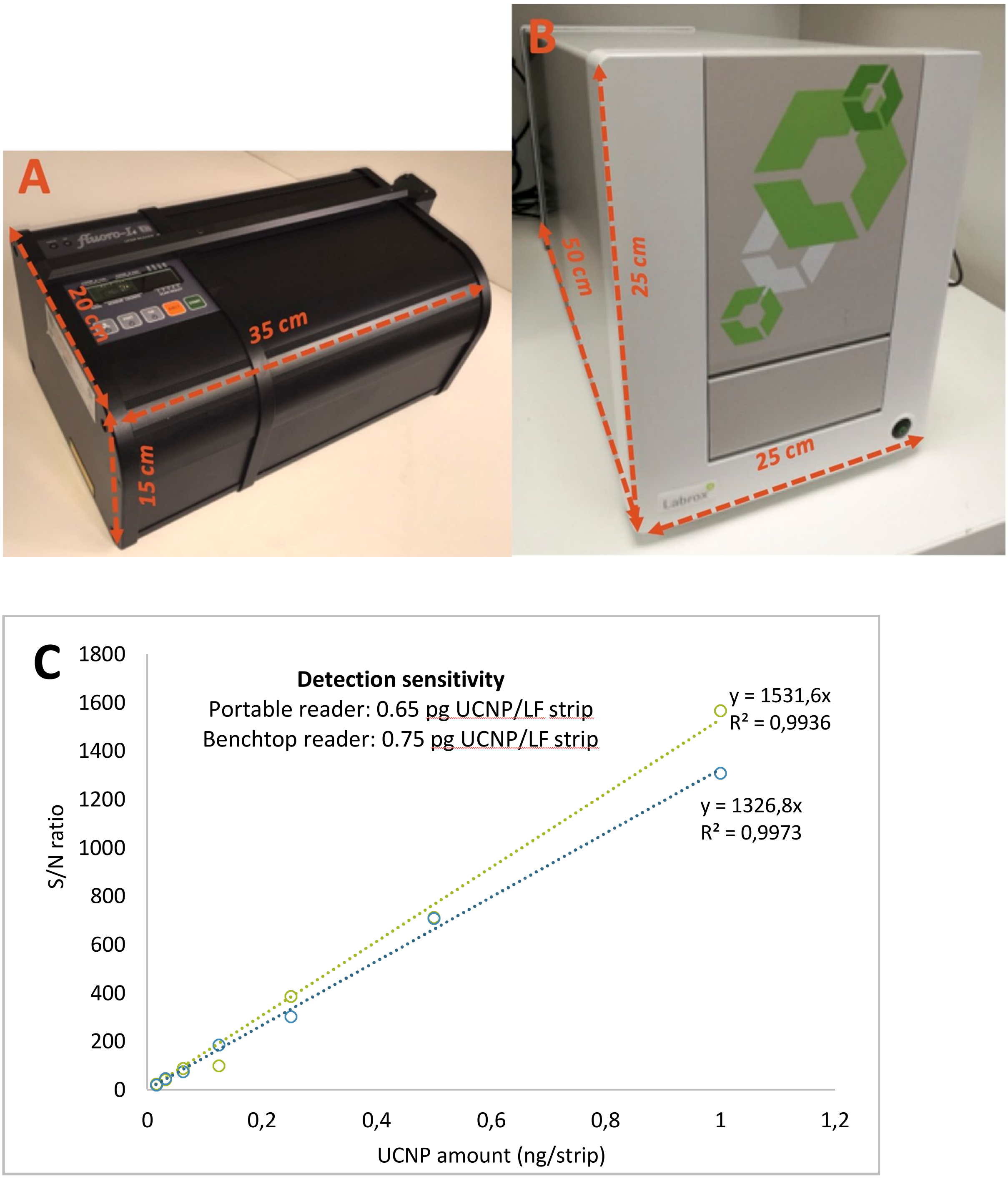


**Fig. S10** Comparison two UCNP readers. (A) Battery powered portable UCNP reader device (DesignInnova, New Delhi, India); (B) Benchtop UCNP reader device (Labrox Oy, Turku, Finland). (C) Comparison of readers for UCNP detection sensitivities. Non-conjugated UCNP particles were striped on the NC membrane. Noise level was determined as average of the device background plus 3 times standard deviation of the device background. UCNP detection sensitivities 0.65 pg UCNP per 5 mm strip for the portable reader and 0.75 pg UCNP per 5 mm strip for the benchtop reader were calculated from the equations of the curves

## HBsAg 0 IU/mL

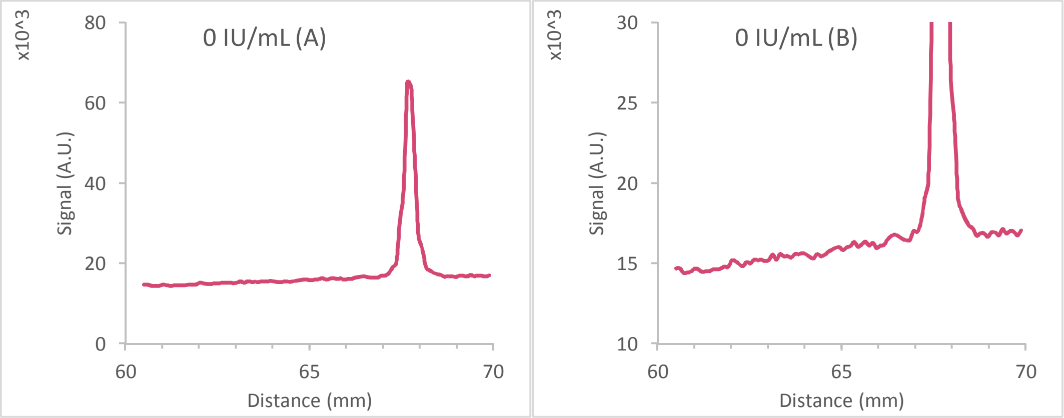


## HBsAg 0.01 IU/mL

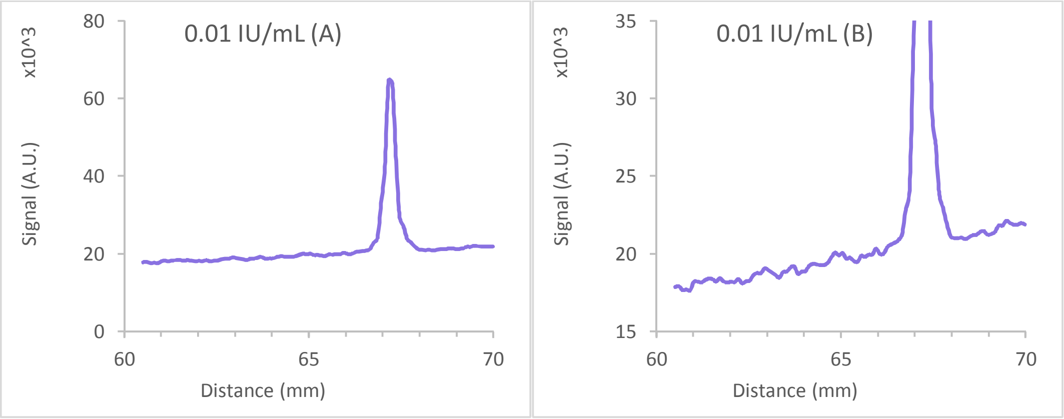


## HBsAg 0.05 IU/mL

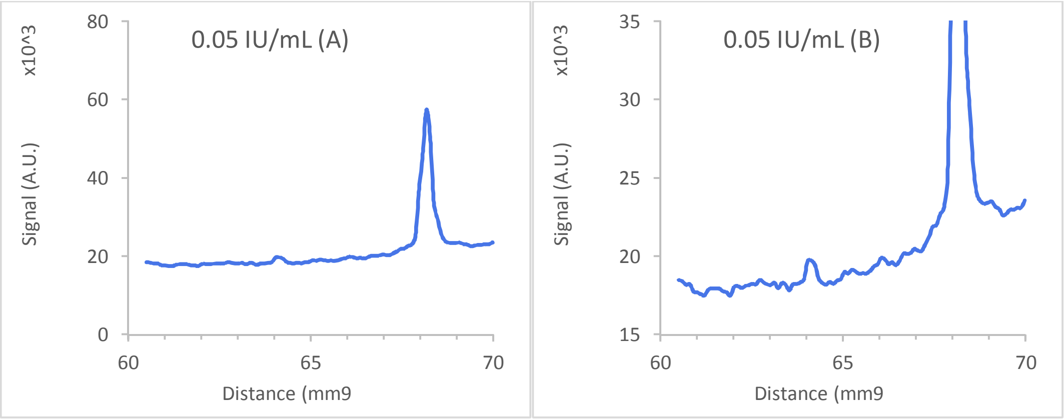

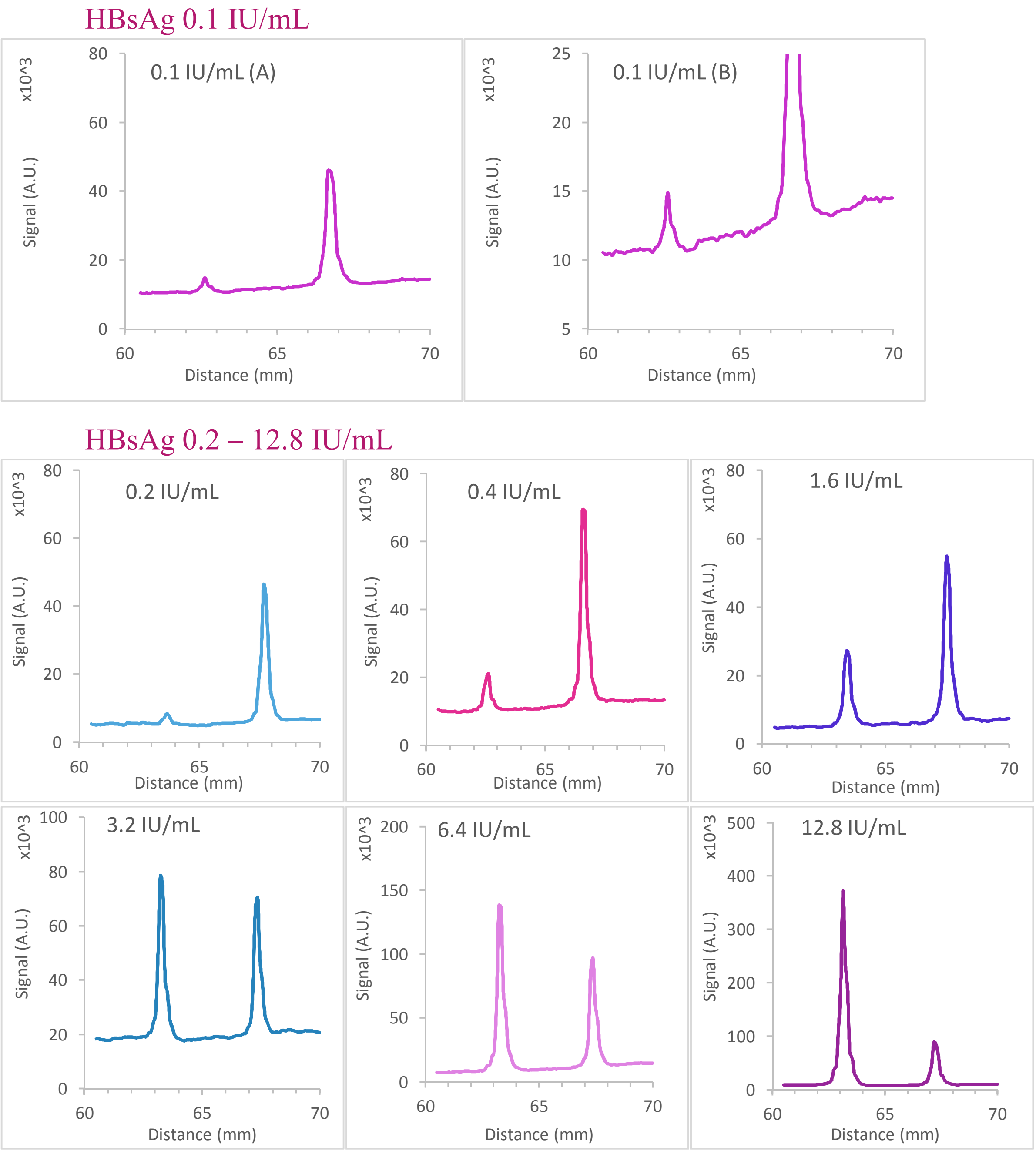


**Fig. S11** UCNP-LFIA strip profiles from the LoD determination experiment using serum spiked with the WHO International Standard (concentration range of 0 – 12.8 IU/mL). The strip profile images show the distribution of detected photoluminescence over the length of the nitrocellulose membrane. The test and control line locations are approximately at 63-65 mm and 67-70 mm from the beginning of the strip, respectively. The strip profiles for HBsAg concentrations 0, 0.01, 0.05, and 0.1 IU/mL (one representative image per concentration) are shown in full-scale figures (A) and close-up figures (B). For HBsAg concentrations of 0.2 – 12.8 IU/mL (one representative image per concentration), only full-scale figures are shown. There is a slight increase in the strip baseline signal from left to right, which is because we have read the strip in the wet form (30 minutes from the addition of samples), and some particles may be still moving towards the absorbent pad (right side). However, this difference in the baseline signal along the strip's length does not affect the test result as we subtract the baseline signal from both sides of the peak
